# Supplementary material for: Anomalous water molecular gating from atomic-scale graphene capillaries for precise and ultrafast molecular sieving
Source: Nat Commun. 2023 Oct 19;14:6615. doi: 10.1038/s41467-023-42401-4 (PMC10587158; doi:10.1038/s41467-023-42401-4)
Supplement: Supplementary file 1 — Supplementary Information [file 41467_2023_42401_MOESM1_ESM.pdf]

Supplementary Information

Anomalous Water Molecular Gating from Atomic-scale Graphene Capillaries for Precise and Ultrafast Molecular Sieving

Qian Zhang†, Bo Gao†, Ling Zhang†, Xiaopeng Liu, Jixiang Cui, Haiyun Che, Hongbo Zeng*, Qun Xu*, Xinwei Cui*, Lei Jiang

* E-mails: xinweic@zzu.edu.cn; qunxu@zzu.edu.cn; hongbo.zeng@ualberta.ca.

† These authors contribute equally to this work.

Supplementary Figures

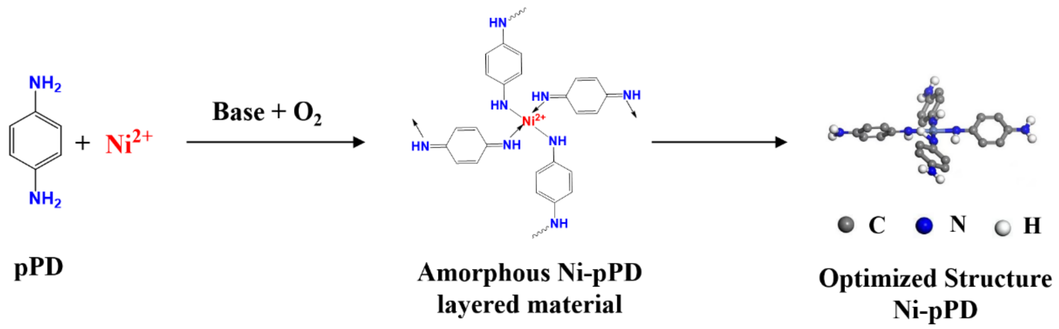

Supplementary Figure 1| Synthesis route of an amorphous, metal-organic layered material, Ni-pPD, with the opmtized structure computed by DFT.

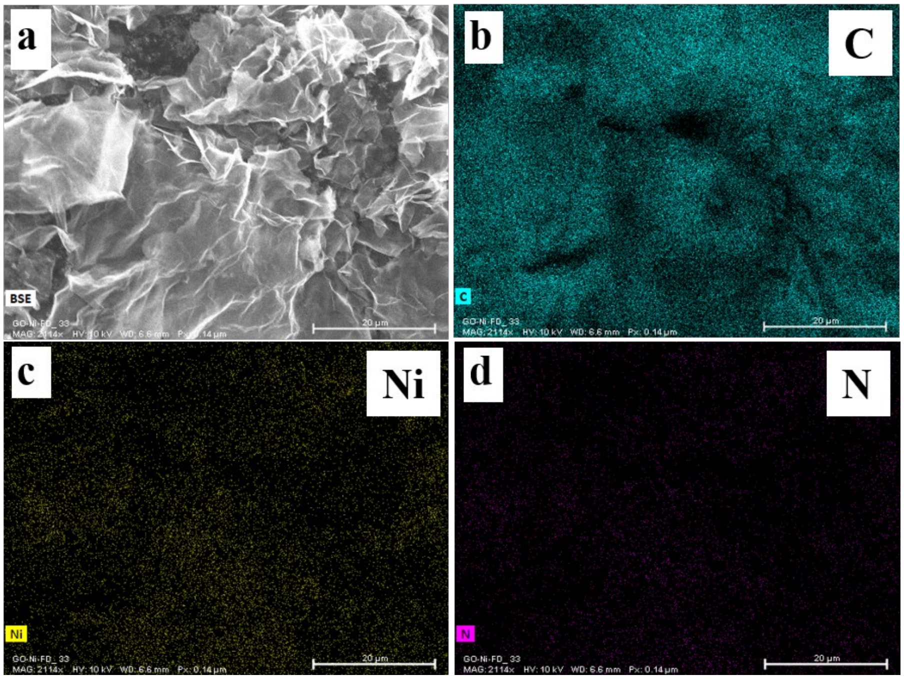

Supplementary Figure 2| SEM analyses of Ni-pPD@rGO nanosheets. **a**, Mophology of Ni-pPD@rGO nanosheets. **b**,**c**,**d**, EDX mapping of Ni-pPD@rGO nanosheets showing the homogeneous distribution of C, Ni, and N.

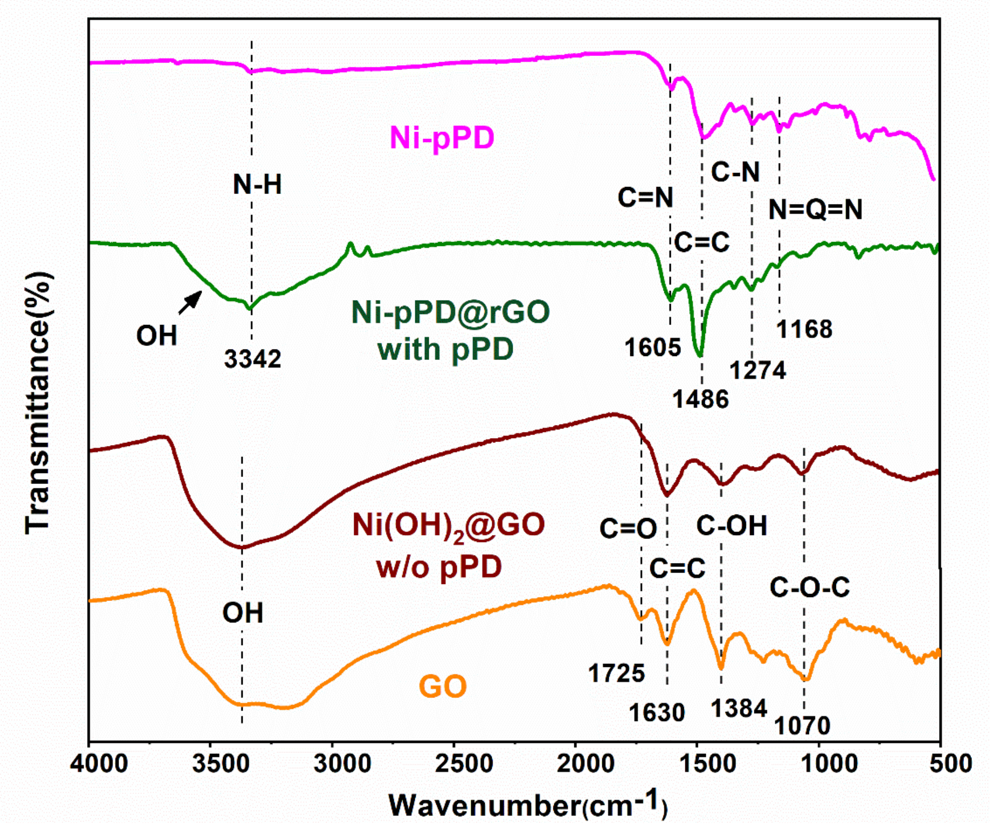

Supplementary Figure 3| FTIR spectra of GO, $Ni(OH)_2$@GO without adding pPD, Ni-pPD@rGO nanosheets with adding pPD, pure Ni-pPD.

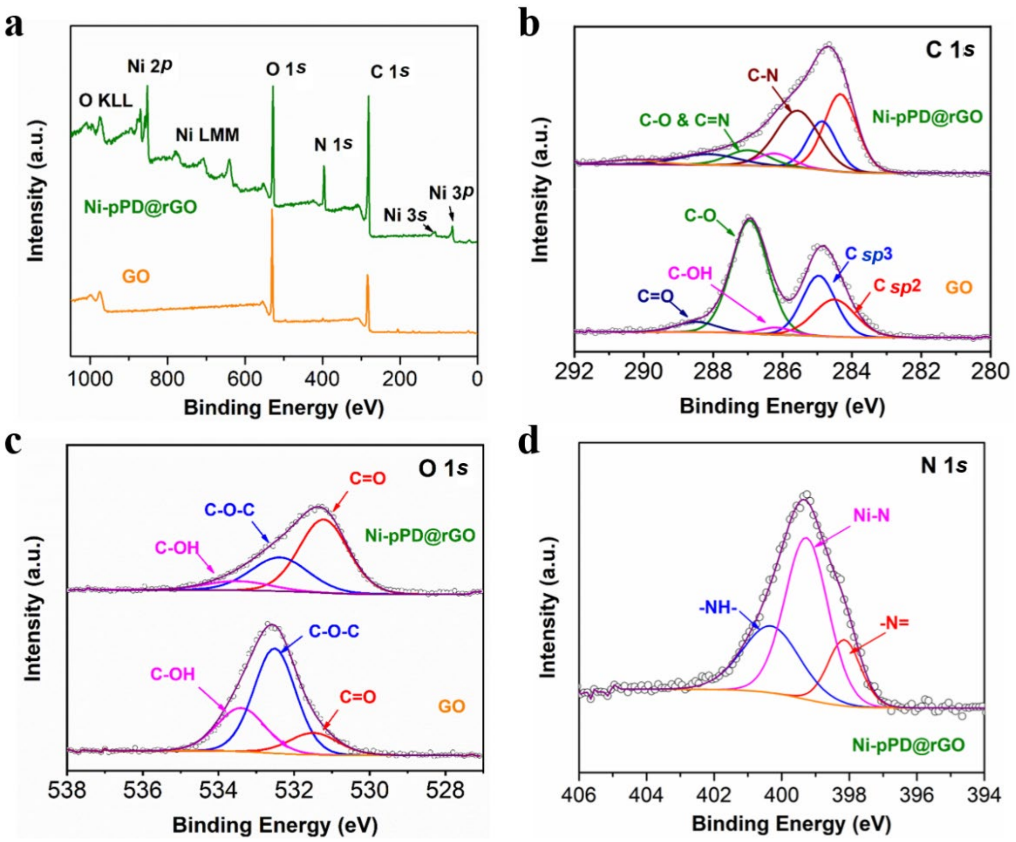

Supplementary Figure 4| XPS analyses of Ni-pPD@rGO nanosheets in comparison with GO nanosheets. **a**, Survey spectrum. **b**,**c**,**d**, High resolution XPS of C 1*s*, O 1*s*, and N 1*s*.

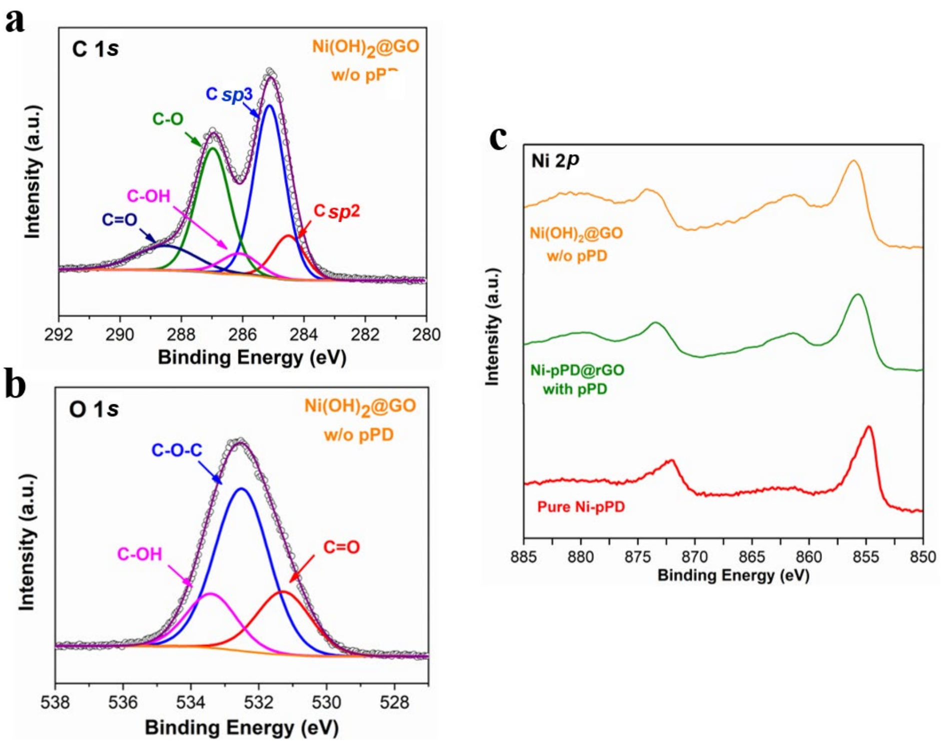

Supplementary Figure 5| XPS analyses of $Ni(OH)_2$@GO nanosheets prepared by GO treated with $Ni(NO_3)_2$ and NH_4OH without adding pPD. **a**, C 1*s*. **b**, O1*s*. **c**, Ni 2*p* in comparion with Ni-pPD@rGO nanosheets and pure Ni-pPD layered particles. No reduction of GO occurred without adding pPD in the synthesis route. Comparing FTIR and XPS results, it is clearly suggested the participation of pPD in the reaction for the formation of Ni-pPD complex, and at the same time, for the reduction of GO to rGO.

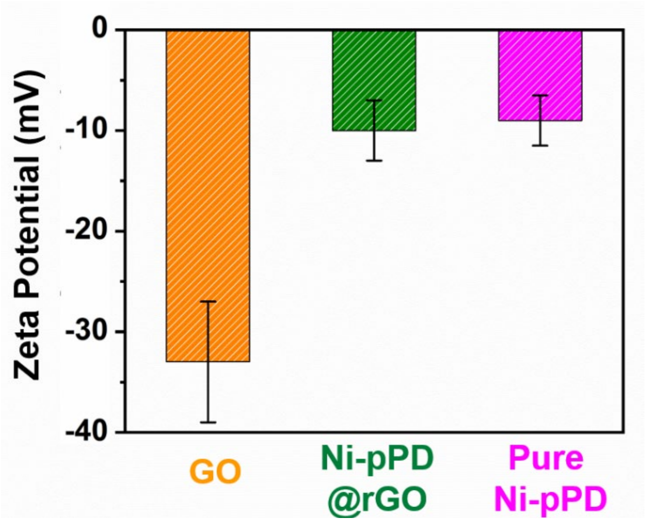

Supplementary Figure 6| Zeta potential analyses of Ni-pPD@rGO nanosheets in comparison with GO nanosheets and pure Ni-pPD particles.

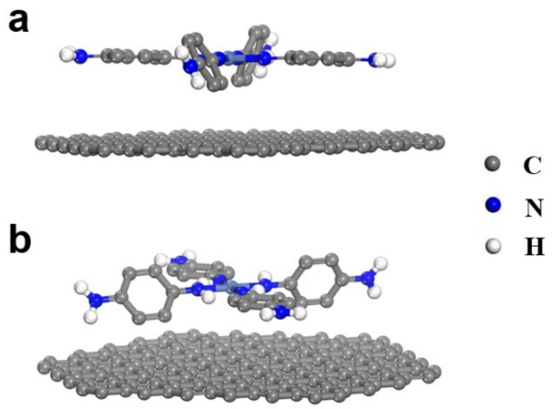

Supplementary Figure 7| The initial structure of Ni-pPD@rGO nanosheets before relaxation by DFT. a,**b**, view from two different angles.

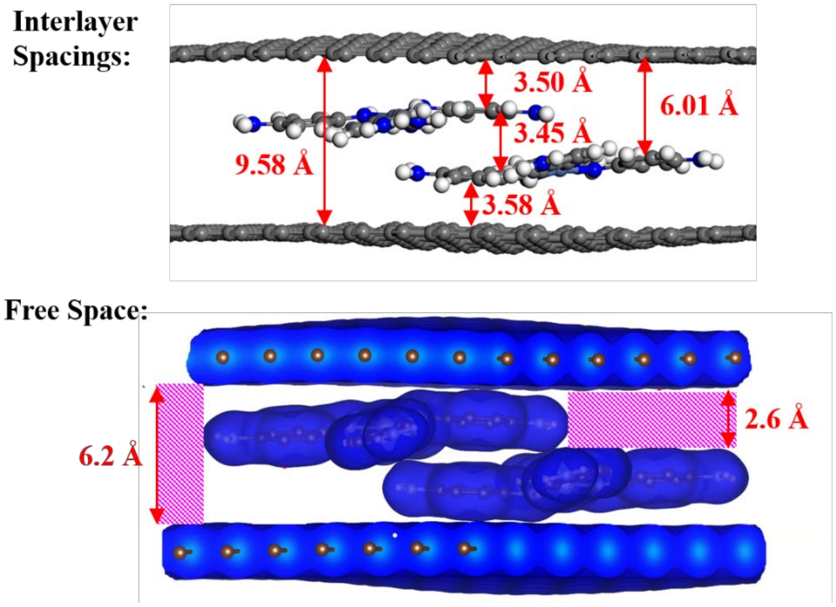

Supplementary Figure 8| Optimized structure of restacked Ni-pPD@rGO nanosheets computed by DFT. π-π stacking formed between graphene and Ni-pPD nano-island, and between two Ni-pPD nano-island, leads to the interlayer spacing between two neighboring graphene being 9.6 Å, which correlates well with HRTEM and XRD results. The restacking results in the free space of 2.6 Å and 6.2 Å for molecules to transport.

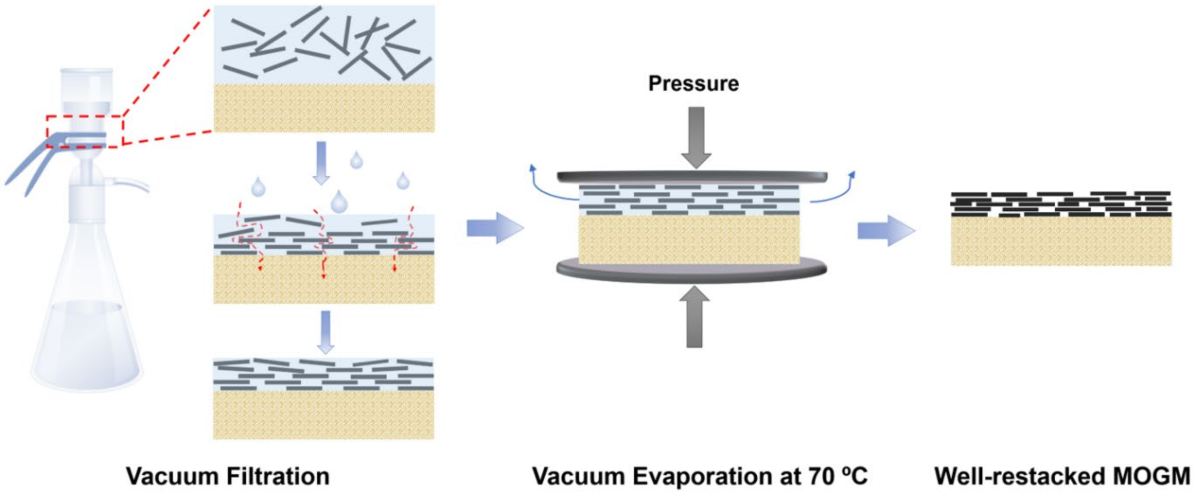

Supplementary Figure 9| Fabrication procedure of MOGMs with well-controlled pressure and temperature during vacuum evaporation.

.

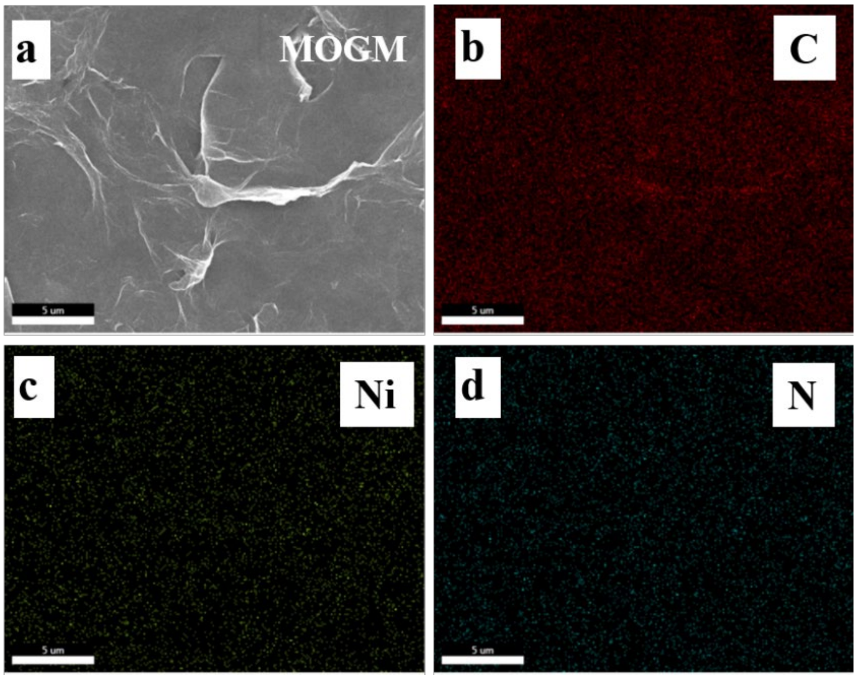

Supplementary Figure 10| Top-view SEM analyses of a MOGM. **a**, Mophology of MOGM. **b**,**c**,**d**, EDX mapping of MOGM showing the homogeneous distribution of C, Ni, and N.

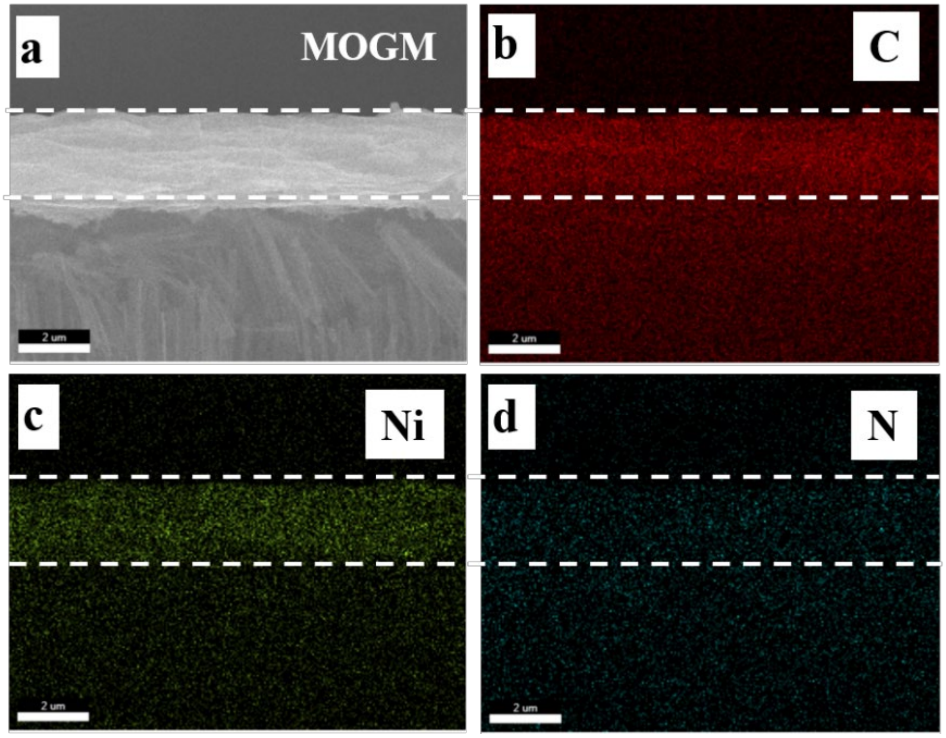

Supplementary Figure 11| Cross-sectional SEM analyses of a MOGM. **a**, Mophology of MOGM. **b**,**c**,**d**, EDX mapping of MOGM showing the homogeneous distribution of C, Ni, and N.

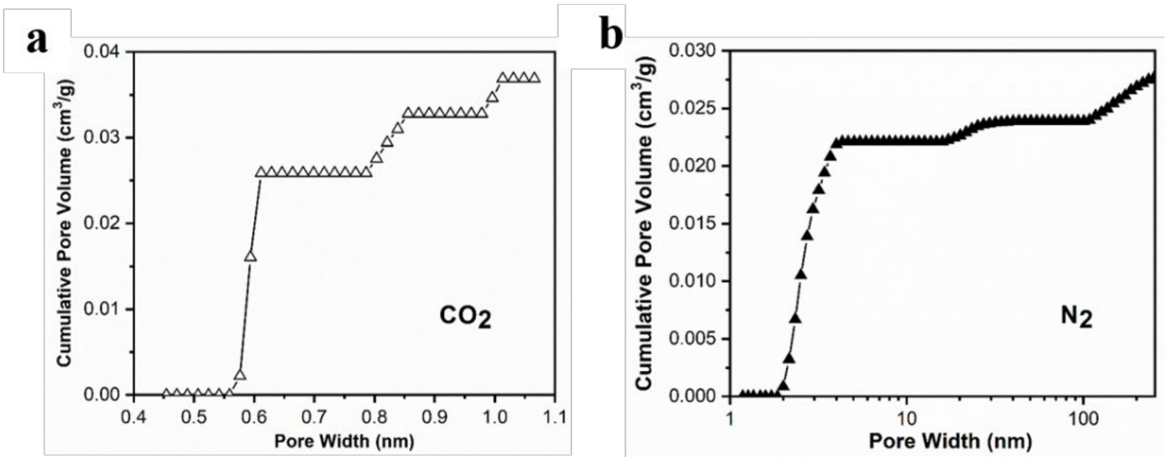

Supplementary Figure 12| BET and PSD analyses of freestanding MOGMs. a,**b**, Cumulative pore volume for CO_2 (**a**) and for N_2 (**b**), both calculated by using a slit pore NLDFT model.

Supplementary Note 1: The strongest peak sits at the pore size of 6.0 Å, corresponding well with XRD and the simulation results, although the pores smaller than 4.5 Å cannot be detected by CO_2 gas. Two other peaks below 1 nm in Fig. 2f may imply that some sub-nano graphene capillaries are formed with three or four layers of Ni-pPD nano-islands sandwiched between two rGO nanosheets. However, because those peaks are relatively weak compared with the main peak at 6.0 Å, it is confident that most nano-islands formed on rGO nanosheets are monolayer Ni-pPD. Also, because the smallest pore size is the determining factor for molecular transport through the macroscopic-scale membranes, the discussion and conclusions made in the main text would not be affected by the pores with larger sub-nano sizes. In addition, the pore size at around 2.5 nm should be considered as the interedge pores between two separated Ni-pPD@rGO nanosheets aligned side by side[1].

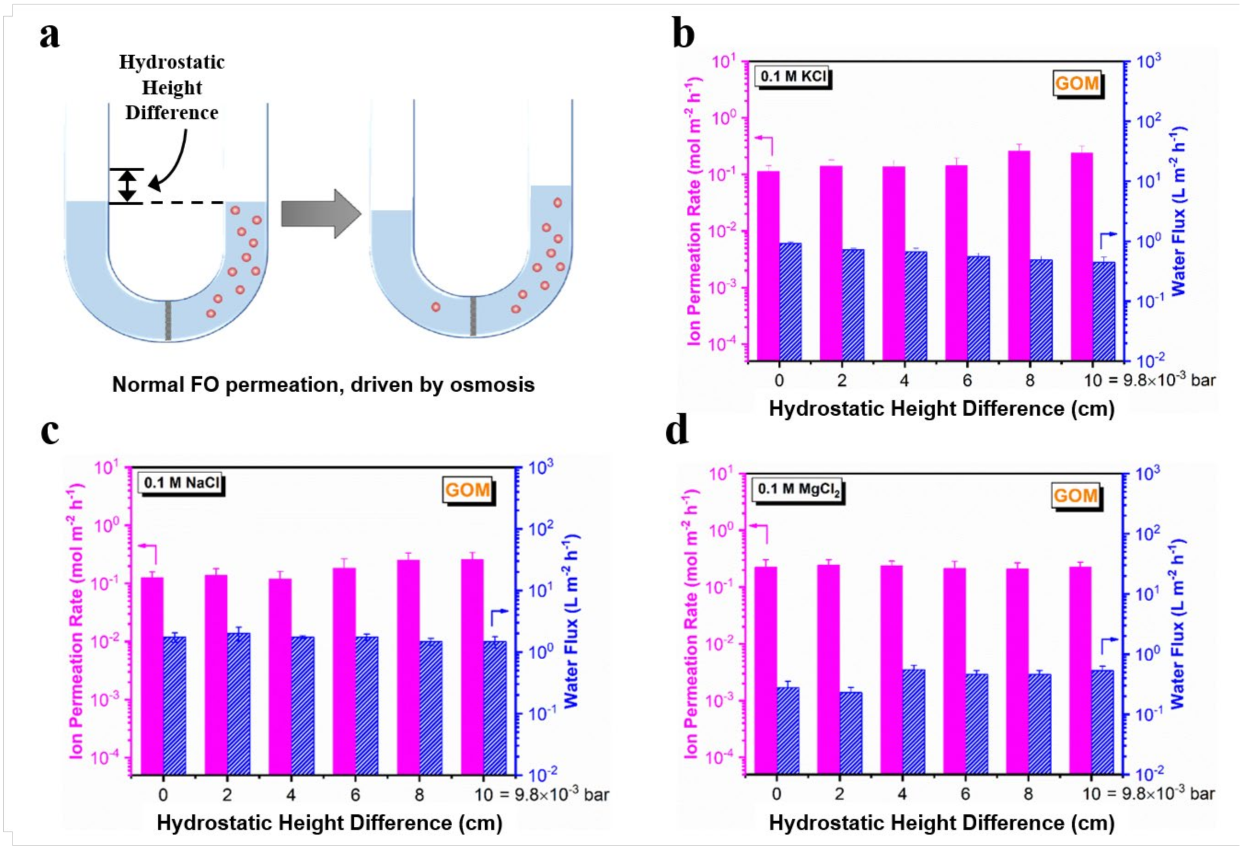

Supplementary Figure 13| Normal FO permeation behavior tested on 750 nm-thick GOMs under the salt concentration of 0.1 M on the draw side. **a**, Normal FO permeation phenomenon driven by osmosis. **b**, 0.1 M KCl. **c**, 0.1 M NaCl. **d**, 0.1 M $MgCl_2$. It is seen that the change of hydrostatic height differences will not affect the ion permeation performance appreciably for GOMs. The change of hydrostatic height differences in the centimeter range is used to conduct fair comparison with MOGMs.

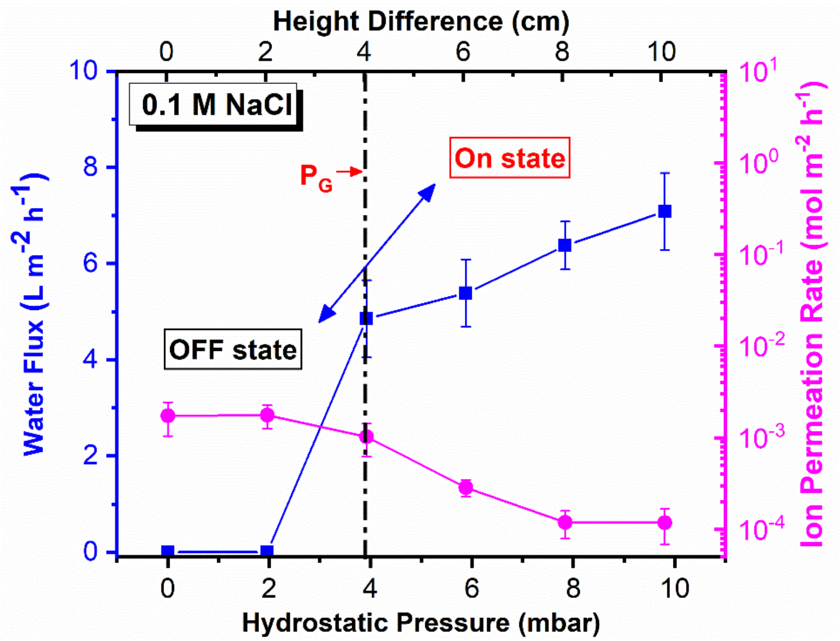

Supplementary Figure 14| Permeation performance of MOGMs in the case of 0.1 M NaCl as the draw solution. **a**, Change of water flux and **b**, ion permeation rates with hydrostatic height differences in the centimeter range.

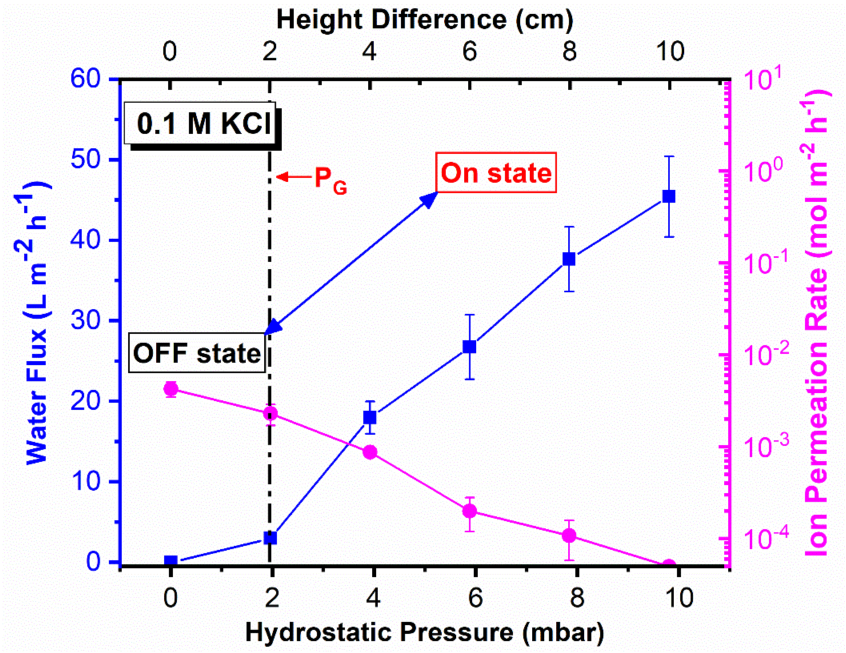

Supplementary Figure 15| Permeation performance of MOGMs in the case of 0.1 M KCl as the draw solution. a, Change of water flux and **b**, ion permeation rates with hydrostatic height differences in the centimeter range.

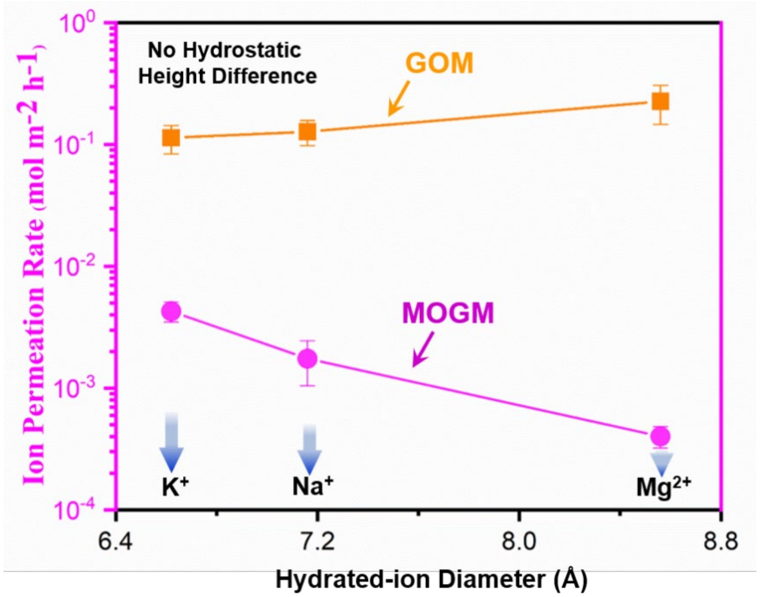

Supplementary Figure 16| Comparison of ion permeation performance of GOM and MOGM in the case of 0.1 M KCl, 0.1 M NaCl, and 0.1 M $MgCl_2$ as the draw solutions, when no hydrostatic pressure is applied. It is noted that ion permeation rate increases with the diameter of hydrated ions in GOMs, driven by osmotic pressure. However, the ion permeation rate decreases with the diameter of hydrated ions in MOGMs, which implies that size exclusion effect plays a key role in this case and partial dehydration of ions should occur when permeating through MOGMs[2].

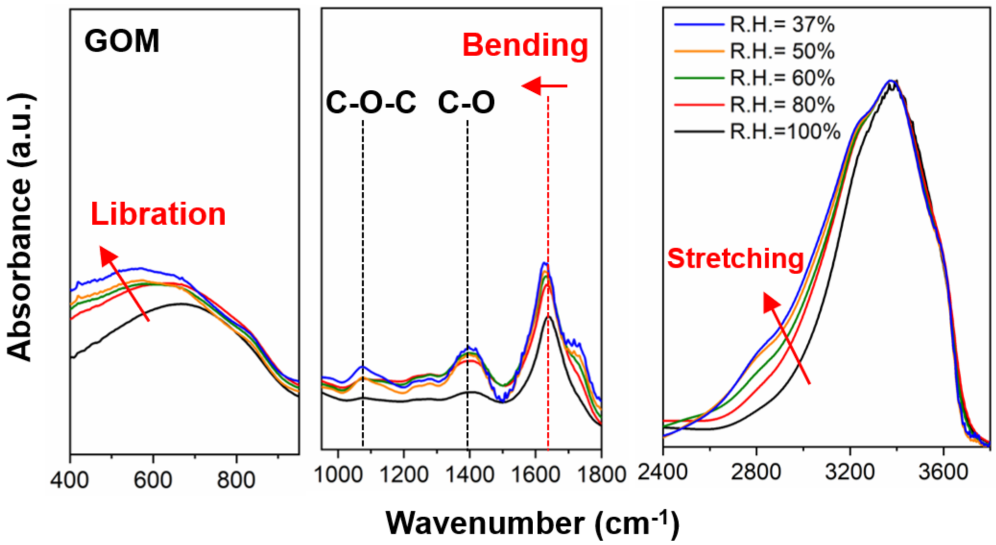

Supplementary Figure 17| In-situ FTIR for the absorbance of water inside GOMs from 37% to 100% of relative humidity. Water content increases from top to bottom. Three different spectral ranges are shown to highlight vibrational modes of intercalated or adsorbed water. The red shift of H_2O bands with decreasing humidity indicates that H-bonded water is stronger within the atomic-scale graphene capillaries in GOMs than that in bulk liquid water. However, due to the inferior anti-swelling properties and the hydrogen-bonding interaction between H_2O and oxygenated functional groups on the hydrophilic walls, the liquid-gating mechanism has not been found for GOMs.

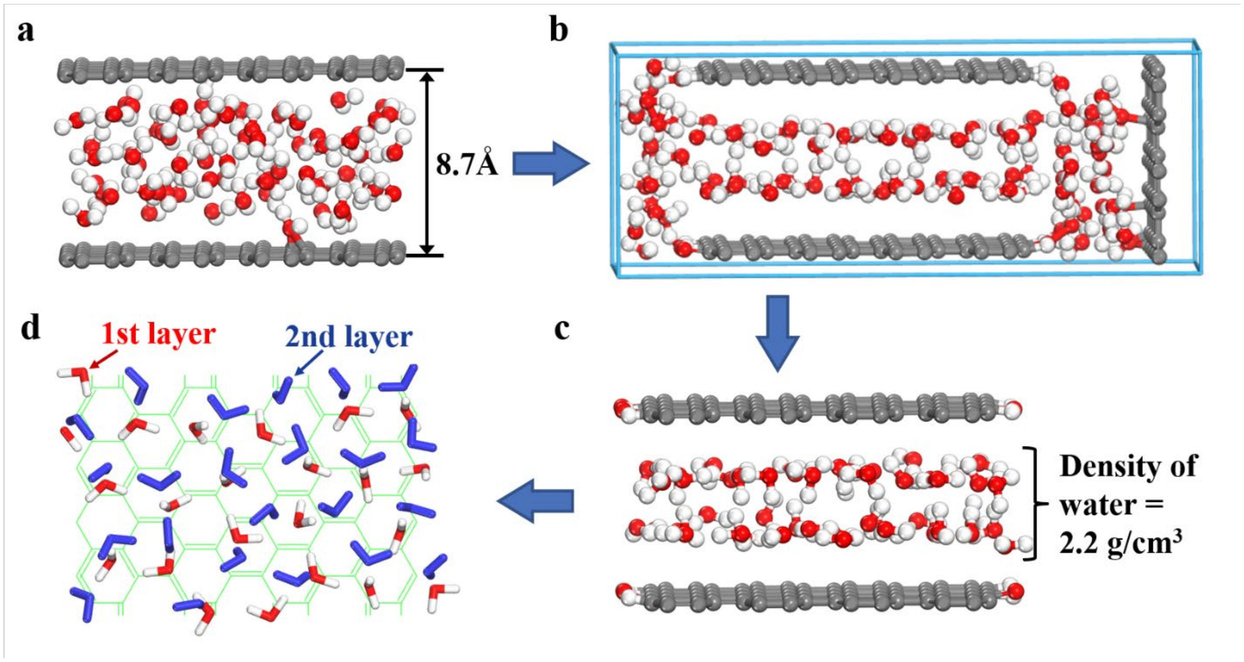

Supplementary Figure 18| DFT calculations of the arrangment of water molecules within the graphene capillaries with the interspacing of 8.7 Å. a, The initial state before optimization. **b**, The final state of the configuration after optimization. **c**,**d**, Density of water and double-layer, hexagonally-arranged water molecules within the highly-confined graphen space.

Supplementary Note 2: In Fig. 4c, a graphene capillary with the interspacing of 6.0 Å was constructed, using the rectangular graphene structure composed of 72 carbon atoms (grey atoms) as the basic unit. 45 water molecules (red: oxygen atoms; white: hydrogen atoms) were sandwiched between them as the initial state. In addition, another graphene nanosheet was positioned vertically to divide the periodic box into two equal-volume compartments. After relaxation, some water molecules diffused out of the highly confined space to the compartments, leaving a highly-compact, monolayer, quasi-crystal water within the sub-nano capillary, with the density around 2.2 g/cm^3 and the unit cell in a hexagonal shape. The density of water in the outside compartments after optimization is 1.0 g/cm^3, further suggesting the validity of the computation.

In Supplementary Fig. 198, a graphene capillary with the interspacing of 8.7 Å was constructed, 90 water molecules were sandwiched between them as the initial state (**a**). After relaxation, some water molecules diffused out of the highly confined space to the compartments (**b**), leaving a double-layer, quasi-crystal water formed within the sub-nano capillary, with the density still around 2.2 g/cm^3 (**c**) and the unit cell in a hexagonal shape in both the first and the second layers (blue) of water nanosheets (**d**). The density of water in the compartments outside is 1.1 g/cm^3. From the direction of individual water molecules, it is easily seen that water molecules are highly correlated by hydrogen bonding, either within the individual layer or between the two layers.

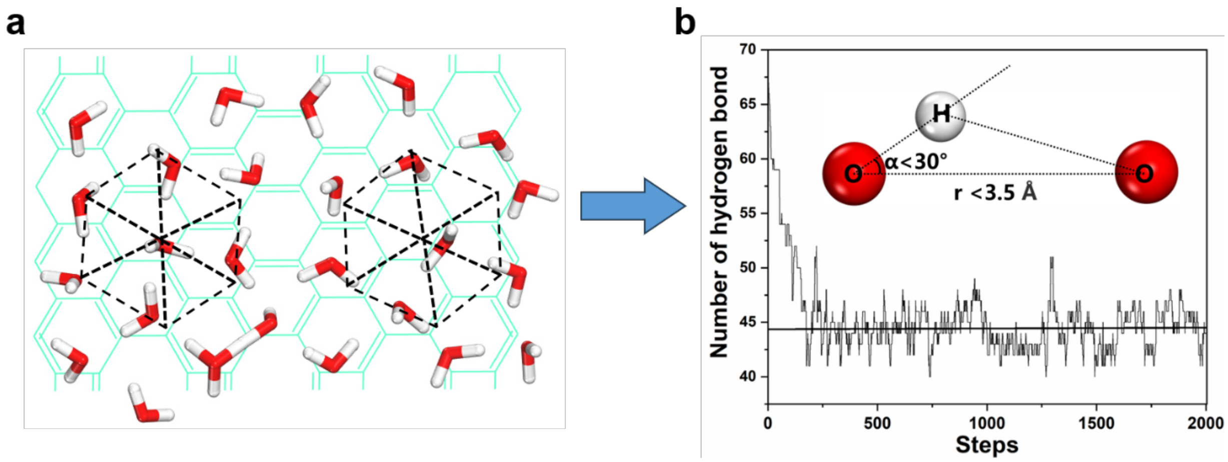

Supplementary Figure 19| Quantitative determination of the average number of hydrogen bonds around each water molecule, using VMD software. The definition for a hydrogen-bond is that the distance between two oxygen atoms $R_{o\text{-}o}$ < 3.5 Å and α < 30° [3]. Putting these criteria into VMD software, the average number of hydrogen bonds for the water structure in **a** can be calculated to be 44 in **b**. Considering the hexagonal type of unit cell, there are around 18.2 water molecules in **a**. In addition, each hydrogen bond is shared by two water molecules, and thus, there are averagely 4.83 hydrogen bonds around each water molecule (HB·H_2O = 4.83).

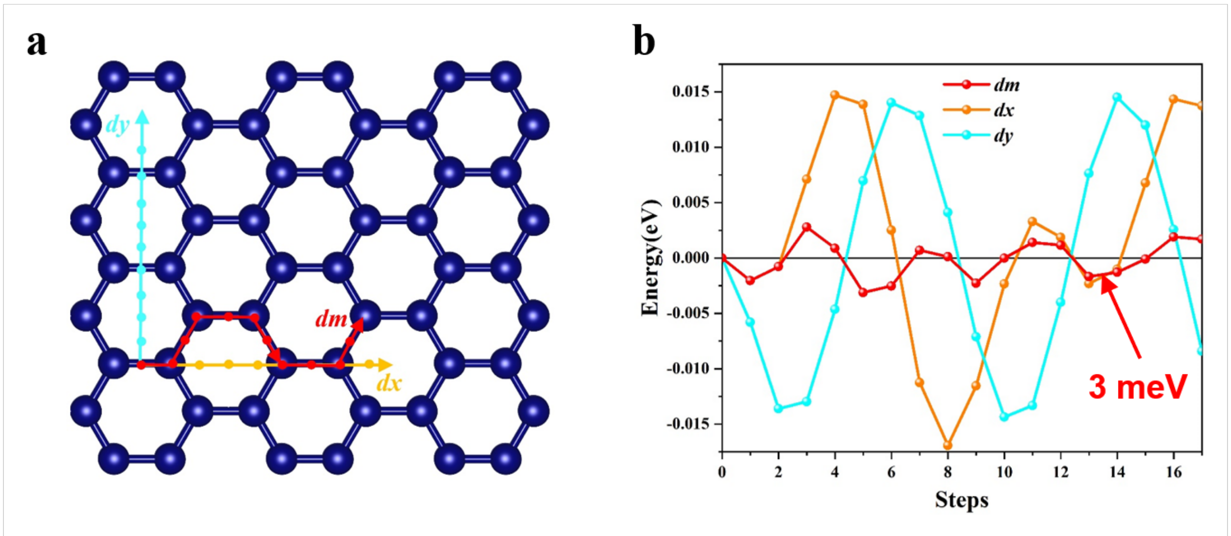

Supplementary Figure 20| Energy barriers of a water molecule moving between two graphene nanosheets with the interspacing of 6.0 Å. a, Three transport routes. **b**, Energy barriers along three transport routes.

Supplementary Note 3: We employed a rectangular unit cell with 54 carbon atoms in each graphene layer, the interspacing of 6.0 Å, and the same periodic boundary conditions as those presented in Supplementary Fig. 18. Between two graphene nanosheets, a water molecule traverses 17 steps in three separate directions, with a step size of roughly 0.355 Å. Each point in **b** is the system energy calculated, while the energy barrier can be obtained from the maximum energy value along the designated transport route.

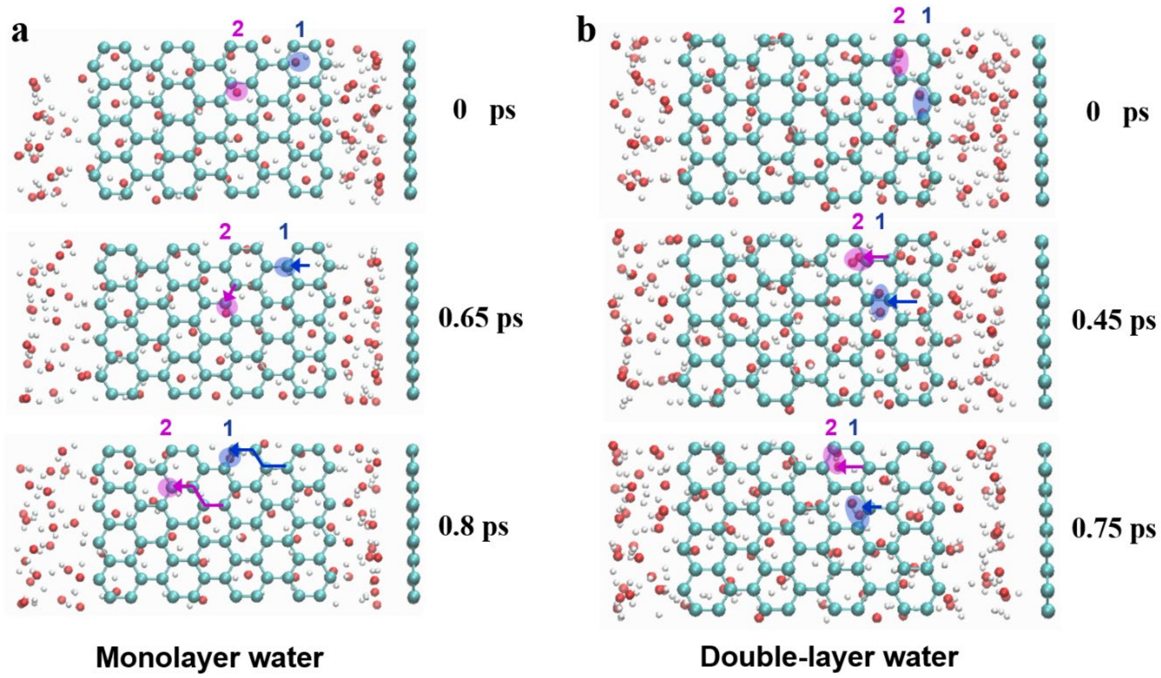

Supplementary Figure 21| MD simulation of bulk movement of water nanosheet(s) within the highly-confined graphene space. a, Three sequential snapshots of MD simulation for the bulk flow of the monolayer water nanosheet (See Supplementary Movie 3). The interspacing of the graphene capillary is 6.0 Å. **b**, Three sequential snapshots of MD simulation for the bulk flow of the double-layer water nanosheet (See Supplementary Movie 4). The interspacing of the graphene capillary is 8.7 Å.

Supplementary Note 4: The initial water density in the compartment on the left is 1.0 g/cm^3, and that on the right is 1.6 g/cm^3. After the bulk movement of water molecules till the equilibrium state, the water density at two compartments reaches the same value (1.3 g/cm^3). Therefore, it also implies that a small pressure difference would generate the bulk flow of water molecules through the sub-nano graphene capillaries, justifying the reliability of our MD simulations .

Supplementary Fig. 21 clearly demonstrates that the bulk movement of monolayer and double-layer water nanosheets follow two different paths under the same conditions, which is more obvious in supplementary movies 3 and 4. In (**a**), the monolayer water nanosheet move along C-C bonds following zigzag directions. In (**b**), however, the double layer water nanosheets move directly across the empty space of the benzene rings. Since the transport route in (**a**) has a lower energy barrier than that in (**b**), as indicated by supplementary Fig. 20, it means that the energy barrier for the bulk movement of double-layer water nanosheets is smaller than that for the monolayer water nanosheet.

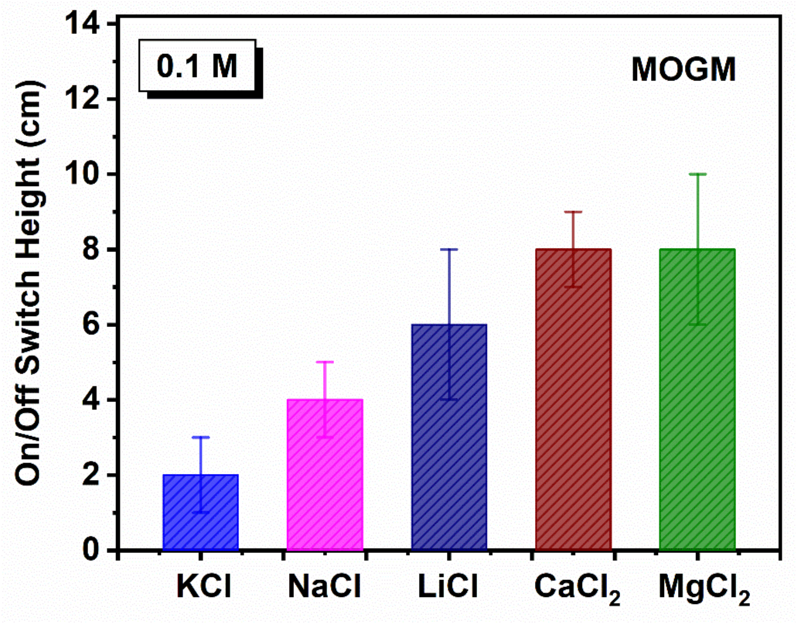

Supplementary Figure 22| Different on/off switch heights observed for various ions, including K^+, Na^+, Li^+, Ca^{2+}, and Mg^{2+}.

Supplementary Note 5: When extrapolating the water flux data to zero applied hydrostatic pressure, Supplementary Fig. 15 shows zero LMH for 0.1 M KCl draw solution, while Supplementary Fig. 14 shows 4 LMH for 0.1 M NaCl draw solution. This observation means that the slope of the water flux data in the "ON" state is smaller for the case of 0.1 M NaCl than that for the case of 0.1 M KCl. This is also another experimental proof that the resistance for the sliding of the 2D water nanosheet to the draw (salt side) is dependent on the type of the draw solutions.

According to the MD simulation results in Fig. 5a and supplementary Movie 1, ions have tendency of hitting the wall at the solid water/liquid water interface, due to the random motion of ions under diffusion outside of the graphene capillary. Hence, larger hydrated ions should exert higher resistance to the sliding of the water nanosheet to the draw side (salt). It may explain that the on/off switch heights are different for different ions, although the differences are small.

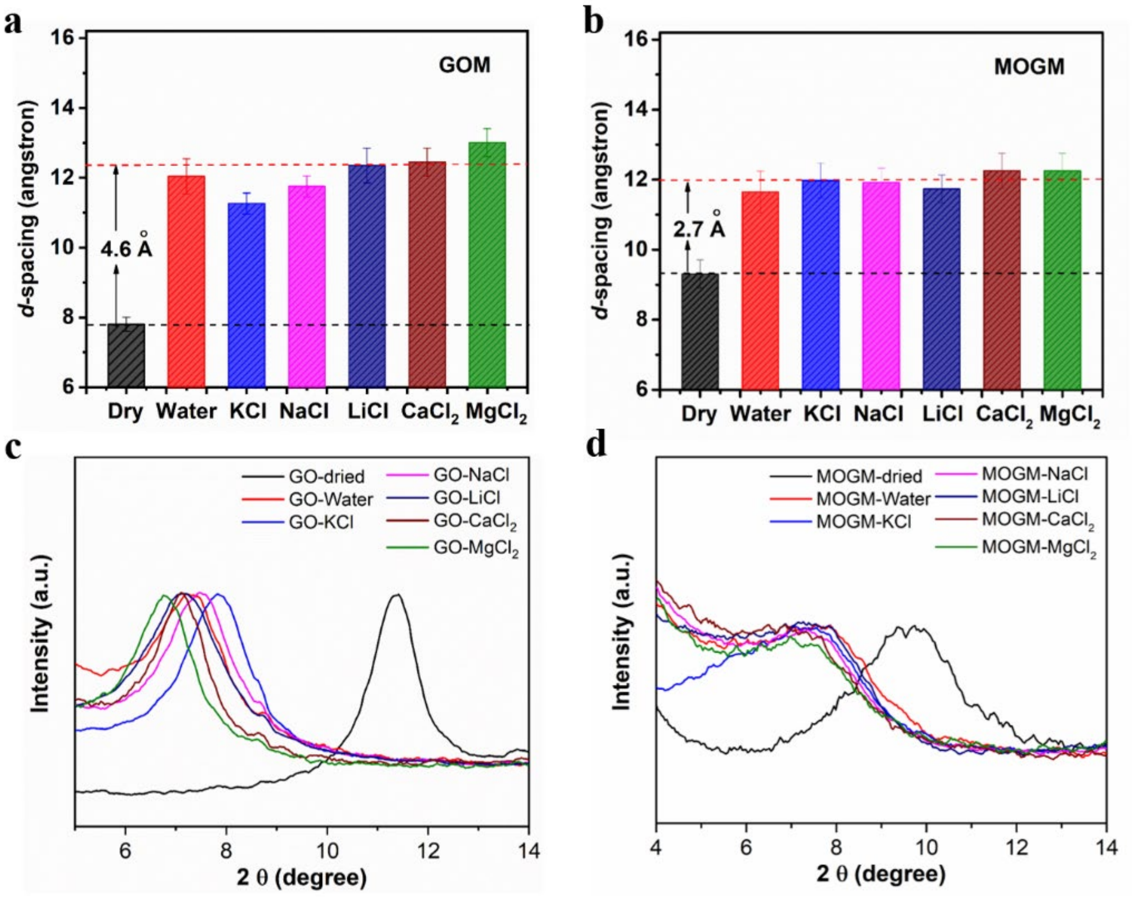

Supplementary Figure 23| Anti-swelling properties of MOGMs and GOMs. **a**, Change of interlayer spacing of GOMs between the dried membrane and the membranes after merging in 0.1 M various solutions for 14 days. **b**, Change of interlayer spacing of MOGMs between the dried membrane and the membranes after merging in 0.1 M various solutions for 14 days. **c**,**d**, Corresponding XRD spectra for GOMs and MOGMs in (**a**) and (**b**), respectively.

Supplementary Note 6: It is clearly shown in supplementary Fig. 23 that the swelling of MOGMs (2.7 Å) is much smaller than that (4.6 Å) of GOMs. 1.2 Å interlayer spacing means 0.86 Å free spacing for the permeation after swelling. In terms of the initial free space of 2.6 Å, it would be 5.3 Å free spacing for the permeation after swelling.

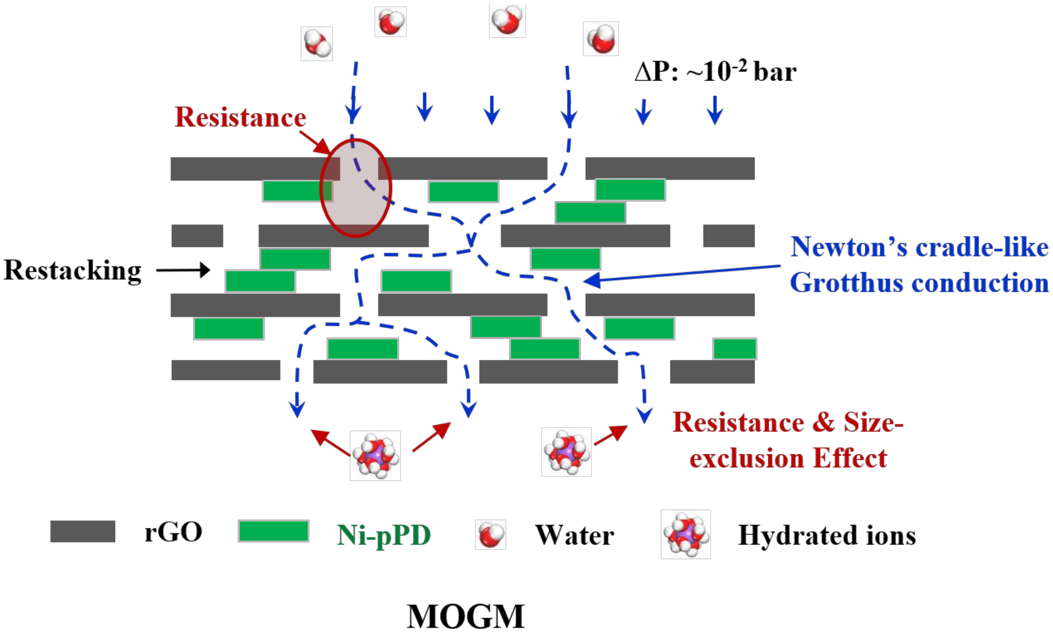

Supplementary Figure 24| Illustration of the origin of the anomalous permeation behavior, including the origin of ultrafast water flux (Newton's cradle-like Grotthus conduction), the highly suppressed ion diffusion (size-exclusion effect), as well as the gating pressures (determined by the magnitude of the resistance from the microscopic defects in the membrane in the figure).

Supplementary Note 7: Although there may be remaining oxygenated functional groups on rGO and traces of $Ni(OH)_2$ present, they can be considered as defective and secondary structures within MOGMs. The primary structures in MOGMs remain as atomic-scale graphene capillaries. As long as the membrane is sufficiently thick, these defective structures cannot form a continuous pathway. Therefore, the atomic-scale graphene capillaries continue to serve as the rate-limiting factor for water and ion transport across the membrane.

Supplementary Table

Supplementary Table 1| Comparison of water flux and ion permeation rates between MOGMs and other 2D material NF membranes reported in the literature in forward osmosis.

2D NF Membrane Design	Thick-ness	Driving Force	Testing Method	Draw Solution	Water Flux (L m^{-2} h^{-1})	Ion Rejection (mol m^{-2} h^{-1})	Ref.
GO Membrane	750 nm	Osmotic Pressure	FO U-shape cell	0.25 M NaCl	0.17	0.18	4
KCl-controlled GO	750 nm	Osmotic Pressure	FO U-shape cell	0.25 M NaCl	0.1	2.7×10^{-4}	4
MXene Membrane	1.1 µm	Osmotic Pressure	FO U-shape cell	0.2 M $MgCl_2$	3	0.035	5
Al^{3+}-intercalated MXene	2.7 µm	Osmotic Pressure (2 M sucrose)	FO U-shape cell	0.1 M NaCl	1.1	4×10^{-3}	5
Ultrathin rGO Membrane	100 nm	Osmotic Pressure	FO Cross-flow test	2 M NaCl	57	0.2	6
GO/rGO	5 µm	Osmotic Pressure (3 M sucrose)	FO U-shape cell	0.1 M NaCl	0.5	0.01	2
GO/rGO	1 µm	Osmotic Pressure (3 M sucrose)	FO U-shape cell	0.1 M NaCl	2.5	0.05	2
GO/UiO66	~ 200 nm	Osmotic Pressure	FO Cross-flow test	2 M NaCl	29.16	0.22	7
MOGM	2.3 µm	Osmotic Pressure (3.9×10^{-3} bar)	FO U-shape cell	0.1 M KCl	18	8.7×10^{-4}	Our work
MOGM	2.3 µm	Osmotic Pressure (5.9×10^{-3} bar)	FO U-shape cell	0.1 M NaCl	4.85	1.0×10^{-3}	Our work
MOGM	2.3 µm	Osmotic Pressure (9.8×10^{-3} bar)	FO U-shape cell	0.1 M $MgCl_2$	2.55	7.3×10^{-5}	Our work

Supplementary References

1. Liu, X. et al. 2D material nanofiltration membranes: from fundamental understandings to rational design. *Adv. Sci.* **8**, 2102493 (2021).
2. Abraham, J. et al. Tunable sieving of ions using graphene oxide membranes. *Nat. Nanotechnol.* **12**, 546–550 (2017).
3. Liu, J., He, X., Zhang, J. Z. H. & Qi, L. W. Hydrogen-bond structure dynamics in bulk water: insights from *ab initio* simulations with coupled cluster theory. *Chem. Sci.* **9**, 2065–2073 (2018).
4. Chen, L. et al. Ion sieving in graphene oxide membranes via cationic control of interlayer spacing, *Nature* **550**, 380–383 (2017).
5. Ding, L. et al. Effective ion sieving with $Ti_3C_2T_x$ MXene membranes for production of drinking water from seawater. *Nat Sustain* **3**, 296–302 (2020).
6. Liu, H. Y., Wang, H. T. & Zhang, X. W. Facile fabrication of freestanding ultrathin reduced graphene oxide membranes for water purification. *Adv. Mater.* **27**, 249–254 (2015).
7. Pang, J. et al. Exploring the sandwich antibacterial membranes based on UiO-66/graphene oxide for forward osmosis performance, *Carbon* **144**, 321–332 (2019).
