# Supplementary material for: Anomalous water molecular gating from atomic-scale graphene capillaries for precise and ultrafast molecular sieving
Source: Nat Commun. 2023 Oct 19;14:6615. doi: 10.1038/s41467-023-42401-4 (PMC10587158; doi:10.1038/s41467-023-42401-4)
Supplement: Supplementary file 3 — Description of Additional Supplementary Files [file 41467_2023_42401_MOESM3_ESM.pdf]

Description of Additional Supplementary Files

Supplementary Movie 1
Description: MD simulation for the "OFF" state under osmosis. The interspacing of the graphene capillary is 6.0 Å. It illustrates the blockage of water and ion diffusion by a monolayer water nanosheet within the highly-confined graphene capillary. Left is pure water and right is NaCl salt solution. Red: oxygen atoms; white: hydrogen atoms; Cyan: graphene sheets; Blue: sodium cations; and Green: chloride anions.

Supplementary Movie 2
Description: MD simulation for the "ON" state under osmosis. The interspacing of the graphene capillary is 6.0 Å. Left is pure water and right is NaCl salt solution. Red: oxygen atoms; white: hydrogen atoms; Cyan: graphene sheets; Blue: sodium cations; and Green: chloride anions.

Supplementary Movie 3
Description: MD simulation for the bulk flow of a monolayer water nanosheet in the "ON" state. The interspacing of the graphene capillary is 6.0 Å. Left has water density of 1.0 g cm^{-3} and right has water density of 1.6 g $^{cm-3.}$ After equilibrium, both compartments have the same water density of 1.3 g $^{cm-3}$. Red: oxygen atoms; white: hydrogen atoms; Cyan: graphene sheets.

Supplementary Movie 4
Description: MD simulation for the bulk flow of double-layer water nanosheets in the "ON" state. The interspacing of the graphene capillary is 8.7 Å. Left has water density of 1.0 g $^{cm-3}$ and right has water density of 1.6 g $^{cm-3}$. After equilibrium, both compartments have the same water density of 1.3 g $^{cm-3}$. Red: oxygen atoms; white: hydrogen atoms; Cyan: graphene sheets.
